# Prof. Silliman

**Published:** 1851-10

**Authors:** 


PROFESSOR SILLIMAN.

The numerous friends and admirers of Professor Silliman, in the west and south will be pleased to learn of his return to the United States, from his European travels. He arrived in the Pacific, and will soon return to Louisville, preparatory to the opening of the session for lectures in the medical department of the University of Louisville. Prof. Silliman's means of chemical instruction were, it is known to those who have been in his classes, ample for a comprehensive course, but he has increased them during his recent visit to Europe. B.

